# A234 PARASITIC INFECTION MIMICKING CROHN'S DISEASE: AN UNUSUAL PRESENTATION OF LOWER GASTROINTESTINAL SCHISTOSOMIASIS

**DOI:** 10.1093/jcag/gwac036.234

**Published:** 2023-03-07

**Authors:** E Vantomme, J Jin

**Affiliations:** Gastroenterology, University of Alberta, Edmonton, Canada

## Abstract

**Background:**

Chronic intestinal schistosomiasis (CIS) is a disease caused in humans by infection with one of seven schistosome species. These species are predominantly found in Africa, South America, and East Asia. Symptoms of CIS include abdominal pain, anorexia, weight loss and diarrhea. In cases of large parasitic burden, overt gastrointestinal bleeding can occur. To our knowledge there are only three other cases of CIS mimicking Crohn's Disease that have been published in the literature. In each of these cases, this was associated with ileal disease, perianal fistulas, or both.

**Purpose:**

We present the first case of CIS in North America that mimicked Crohn's Disease on imaging with no associated ileal or visible perianal disease.

**Method:**

Case report of a patient presenting to a tertiary care hospital in Edmonton, Alberta.

**Result(s):**

A 47 year old man who recently immigrated from Eritrea presented to the emergency department in June 2020 with red blood per rectum. Digital rectum exam revealed small external hemorrhoids. He was discharged with a plan for outpatient abdominal CT scan and colonoscopy. He presented again to the emegency department in January 2021 where he complained of back pain exacerbated by bowel movements productive for small, hard stool. He also began seeing white discharge mixed with his stool. His back pain, white discharge, and blood-streaked bowel movements persisted for over a year, and he developed perianal pain that was exacerbated by valsalva. A CT scan of the abdomen/pelvis revealed extensive soft tissue swelling in the anorectal region, extraluminal gas, and a 15mm fluid collection adjacent to the coccyx. A diagnosis of fistulizing Crohn's Disease was suspected. A digital rectal exam and colonoscopy were performed September 19, 2022 which revealed small hemorrhoids but otherwise endoscopically normal colonic and ileal mucosa. Biopsies from the rectum revealed eosinophilic granulomas associated with parasitic ova; a diagnosis of schistosomiasis was made.

**Image:**

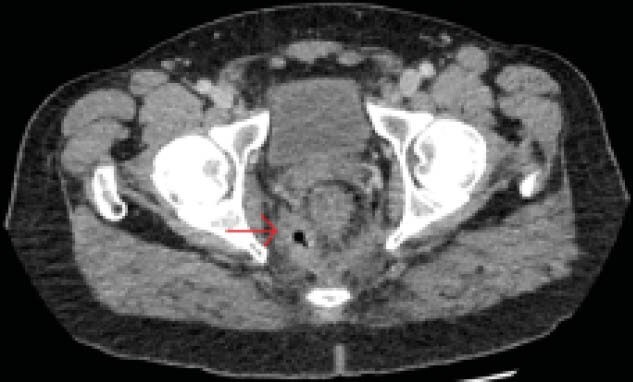

**Conclusion(s):**

CIS is a rare diagnosis in North America. It is unusual for this disease entity to present is a mimic of fistulizing Crohn's Disease. This case report reminds the clinician that a high index of suspicion in patients presenting from endemic regions is required to make this diagnosis.

**Please acknowledge all funding agencies by checking the applicable boxes below:**

None

**Disclosure of Interest:**

None Declared

